# Sampling statistics are like story creation: a network analysis of parent–toddler exploratory play

**DOI:** 10.1098/rstb.2021.0358

**Published:** 2023-02-13

**Authors:** Hadar Karmazyn-Raz, Linda B. Smith

**Affiliations:** Psychological and Brain Sciences, Indiana University, Bloomington, IN 47401, USA

**Keywords:** parent–toddler play, exploration, network analyses, Zipfian distributions, memory

## Abstract

Actions in the world elicit data for learning and do so in a stream of interconnected events. Here, we provide evidence on how toddlers with their parent sample information by acting on toys during exploratory play. We observed 10 min of free-flowing and unconstrained object exploration of by toddlers (mean age 21 months) and parents in a room with many available objects (*n* = 32). Borrowing concepts and measures from the study of narratives, we found that the toy selections are not a string of unrelated events but exhibit a suite of what we call coherence statistics: Zipfian distributions, burstiness and a network structure. We discuss the transient memory processes that underlie the moment-to-moment toy selections that create this coherence and the role of these statistics in the development of abstract and generalizable systems of knowledge.

This article is part of the theme issue ‘Concepts in interaction: social engagement and inner experiences’.

## 1. Introduction

Much of the data in the world is latent as it is unrealized without some direct physical action. Thus, behaviour—actions in the world—samples information for learning. Theorists [1,2] have conjectured that children's free-flowing play, unconstrained by the goal of solving a specific task, provides a model of optimal sampling for emerging knowledge systems. Here, we provide evidence on how toddlers with their parent sample information by choosing toys during an extended period of exploratory play. We show that toy selections are not a string of unrelated events; instead, they exhibit a suite of temporal statistics and transitions from one toy to the next with a structure like that of a coherent narrative. In the discussion, we consider the transient memory processes that influence moment-to-moment toy selections and the role of the resulting coherence statistics in the development of abstract and generalizable systems of knowledge.

### (a) Human-generated events in time

**Figure 1.**
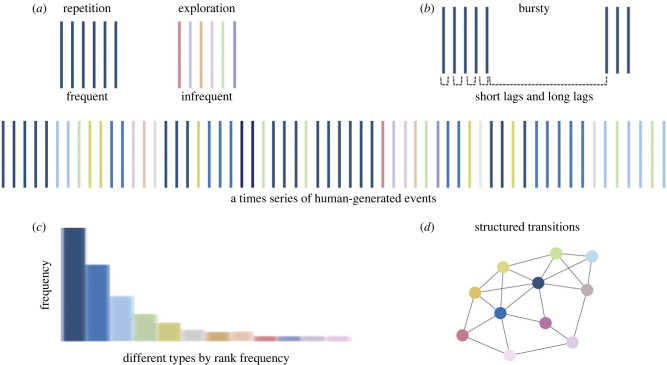
The statistics of human-generated events. The centre figure shows a time series of events with different types of events indicated by different colours. The surrounding figures show different properties of that time series: (*a*) periods of repetition and exploration, (*b*) bursty repetitions (very short or very long delays) of a single type, (*c*) frequency distributions in which some types are very frequent and others infrequent and (*d*) structured transitions among different types with recurring higher order relations. (Online version in colour.)

A considerable body of research across many fields shows that natural time series of human-generated events display a characteristic set of statistical properties [3–11]. In the centre of figure 1 is an illustration of a time series with different types of events shown in different colours. These human-generated events could be the words in a conversation or text [3–5], the places visited on trips across a country [6,7] or the objects encountered as one walks through a home or looks at a series of photographs [8–10]. Or they could be the different toys selected for play by a child in a toy room [11]. Time series of these behaviour-generated events exhibit the statistical properties illustrated in the surrounding panels of figure 1. The temporal structure has periods of repetitions of high-frequency event types and periods of more rare events (*a*). Repetitions of high-frequency items are bursty [4], occurring in clusters of repetitions that recur over long delays (*b*). The temporal patterns yield a skewed-frequency distribution in which a few types are very frequent, but most are infrequent (*c*). Finally, these statistics yield an overall pattern that can be summarized by a small-world [12] network in which a relatively small number of pairwise transitions (one event to the next) connect all the events in the time series (*d*).

In his seminal work on human behaviour, Zipf [13] proposed that highly skewed frequency distributions and burstiness (figure 1*c,b*) are pervasive across human-generated events because they arise from a fundamental property of human motivation, the principle of least effort. Researchers who study how humans sample information have argued that periods of repetitive and varied sampling (figure 1*a*) reflect two opposing human tendencies: exploitation of what is known and exploration of the novel [14–17]. These two tendencies have been linked to ‘curiosity-based’ or ‘predictive’ learning and the idea that learners repeatedly sample from the same source as long as they are learning something new, reducing prediction error, but move to exploration when prediction error is low [18–20]. Other researchers have focused on the bursty repetitions of the same event (figure 1*b*) as an optimal combination of massed and distributed practice and these researchers have shown that this interleaving of a to-be-learned item with other items benefits learning [4,21–23]. Others have focused on relational patterns of co-occurrence and transition (figure 1*d*), often in the context of studying the semantic structure of time series of words or scenes [24–26]. In brief, the suite of statistics that characterize time series of human-generated events are well recognized, often studied independently and known to be relevant to human cognition. However, the field lacks an integrated understanding of the statistics, the degree to which they characterize the everyday experiences of infants and children and the relevance of these statistics to cognitive development. Our specific goal in this study was to quantify these statistics with respect to one everyday experience of toddlers: exploratory play with a mature partner.

### (b) Coherence statistics

What could it mean for cognitive development if the statistics of figure 1 characterized toddlers' experiences during toy play? The study of narratives offers a possible answer. Narratives—conversations, stories—are one kind of human-generated time series that are characterized by the statistics shown in figure 1 [27]. Researchers of narratives, however, have thought about the implications of these statistics through the specific lens of their contribution to forming coherent meaning [28–31]. Coherent narratives are characterized [28,32,33] by a few high-frequency topics (figure 1*c*) that occur in close-in-time repetitions [34] (figure 1*a*) but that are also returned to over longer delays (figure 1*b*). These temporal patterns are believed to connect all the ideas in the narrative into a coherent whole [33,35] (figure 1*d*). Informative narratives have been characterized as ones that also bring in new information interleaved among the more frequent main topics [32,35]. Based on the information-theoretic value of frequent and infrequent information [35], narratives have been characterized as having a Topic–Comment structure. By this information-theoretic definition, ‘Topics’ are high-frequency items that are the ‘given’ information. ‘Comments’ are low-frequency and thus ‘new’ information. Finally, within narratives, the co-occurrences and transitions in time among different contents (figure 1*d*) exhibit higher-order relations [30,31,33]. Experiments (e.g. [23,36,37]) and computational models (e.g. [10,38,39]) show that these statistics benefit communication, integration of new and old information, and learning.

The functions of these statistics identified by theorists of narratives may be the same in learning from human-generated *nonlinguistic* experiences as well as linguistic ones. This is the larger theoretical idea motivating our research program. Here, we take the first step: borrowing the orientation of theorists of narratives [6,7,35], we measure the coherence statistics of parent and toddler toy selection during exploratory play.

### (c) The current study

We asked toddlers along with their parent to freely play in a toy room with many available toys. The parent was included because toddlers are less likely to explore or engage with toys when playing alone and because the company of caregivers is the common context for toddler exploration and learning [40–43]. We defined toy selection as the handling of a toy by the parent or the toddler. Prior work shows that parents often talk about handled toys during toddler play and that this talk has significant effects on toddler behaviour and learning [40–43]. Therefore, we also included parent referential talk as a form of toy selection.

## 2. Method

### (a) Dyads

The participants were 32 toddler–parent dyads; mean age of the toddlers was 21 months (s.d. = 11) and half were female. One- and 2-year-old children were selected for participation because during this time, there is increasing interest in and learning about objects, their uses and their names; further considerable research has linked exploratory play with a mature partner to advances in cognitive development [1,4,41–43]. Data from 16 of the dyads were included in an earlier report of 1-year-old toddlers' experiences of name–object co-occurrences [13]: the sample was broadly representative of Monroe County, Indiana (75% European American, 6% African American, 6% Asian American, 6% Latino and 6% Mixed race) and consisted of predominantly working- and middle-class families. All research was approved by the Human Subjects and Institutional Review Board at Indiana University, Protocol 12603. The predominant language in all homes was English.

### (b) The toy room and instructions

Parents and toddlers were invited to play in a toy room (3 m by 4 m) for 10 min without an experimenter present. At the start of each test session, 32 toys (see electronic supplementary material) were haphazardly distributed on the floor of the playroom. The spatial distribution of toys for each dyad was accomplished by five different experimenters such that the spatial arrangements were purposefully uncontrolled. We used this approach to limit any obvious or repeated organizing principle and to increase the generalizability of our findings to everyday contexts in which the spatial distribution of toys in playrooms is uncontrolled and highly variable.

The 32 toys were selected to be of interest to toddlers but not to strongly prescribe specific goals or kinds of play (e.g. farm play or cooking play.) As a guide to the choice of toys, we used the toys in the toy-box of the family waiting room for the five developmental laboratories at Indiana University. The toys in the waiting room are a happenstance set that emerged organically over time and comprise a variety of both typical and odd items that interest children from infancy to 5 years of age.

Parents were told that we were interested in toddler exploratory play and to encourage their toddler to engage with the toys and that they should interact with their toddler as they normally would. We set the duration of the play session at 10 minutes because pilot studies indicated that this was the maximum period for continually engaged play by toddlers with a parent.

### (c) Audio and video recording

Both the toddler and the parent generated experiences for toddler. Our principal goal, however, was to quantify the statistics of sampled toys *from the young learner's point of view*. Accordingly, we used a head-camera and audio recorder worn by the toddler to record the stream of toy selections from the perspective of the toddler. The lightweight head camera (WATEC model WAT- 230A with WATEC lens model 1920BC-5) had an angle of view of 115.2° on the horizontal and 83.7° on the vertical and was mounted on a headband that could be situated low and firmly on the toddler's head. Children were able to freely move—walk or crawl—while wearing the head camera and commonly did so. Two additional third-person cameras (overhead and from the side) also recorded a full view of the room but were consulted only if there was ambiguity with respect to the identity of an object in a participant's hand.

### (d) Coding

The measured coherence statistics are calculated over *discrete* events. Consistent with established practices in time-series analyses [44], we coded the data in terms of a pre-defined time window. The 5 s window used in this study was chosen based on prior research that directly compared 5 s and 1 s sampling rates of parent and infant behaviour during play and found that the sampling rate did not affect the overall temporal statistics [11].

A coded toy selection consisted of the toy selected and the selection act. There are three possible selection acts: infant handling, parent handling and parent talk. Each coded selection was marked with the timestamp of the 5 s window in which it occurred. Multiple selection events could have the same timestamp: for example, within a single 5 s window, the infant could handle both the cow and the ball, and the parent could name the ball. In this case, the same timestamp would be associated with each of the three selection events. If a unique toy selection act (e.g. toddler handling the cow) bridged two successive 5 s segments, it was marked as two discrete events occurring with the two successive timestamps.

Thus, each toddler's head-camera video and audio were binned into 121 segments of 5 s. Human coders annotated all objects that were in hands during each segment, whose hand and the specific toy, using Datavyu [45]. Trained coders in a separate pass transcribed parent talk within each 5 s segment. From these transcripts, parent talk about specific toys in each segment was determined. Talk referring to a specific toy was defined as any talk that was clearly about a specific object; for example, naming the white sheep as ‘sheep,’ ‘lamb’ or erroneously ‘dog,’ describing the object (e.g. 'he has a lot of white fur', or 'he's soft', 'he's hiding'). Two coders independently coded each video segment for handling and agreed on 90% of the 3872 coded video segments. A third independent coder resolved all discrepancies. Two independent coders transcribed toy references in parent talk within the 5 s segments. The two coders agreed on the referred-to objects on 86% of the 3872 segments and discrepancies were resolved by a third independent coder. Toddler talk was rare, which is expected given the age of the children and the well-known variability in amount of early productive language [44,46]. In addition, early talkers' articulation is highly variable [47,48], making the objective coding of their utterances difficult and reliability poor. Coding reliability could be improved by inferring what the toddler said from parent talk in response to the utterance or from the hand actions of the two participants near in time to the toddler's utterance. However, these behaviours were already being counted as separate acts of toy selection. Therefore, and given the limited amount of toddler talk, the data analyses included only toddler handling, parent handling and parent talk but did not include toddler talk.

### (e) The data structure

The data structure is a time series of discrete toy selection events. Unique selection events consist of the following four components: the selected toy, who did the selection, the selecting behaviour and the timestamp (the segment from 1 to 121 during which the selection occurred). An example of the data from an individual dyad is shown in table 1; we will use this example throughout §3 to explain the different statistical measures and analyses. For all analyses, we set *a priori* the acceptable Type 1 error at *p* < 0.01. The de-identified data submitted for all analyses are available at https://doi.org/10.17605/OSF.IO/HE7TZ.

**Table 1.** The data structure. An example of selection events across three 5 s segments. Individual toy selections are coded in terms of four properties: the selected toy, who did the selection, the act of selection and the timestamp (from 1 to 121) of the 5 s segment during which the selection occurred. Each entry is a unique selection (or data point) because it differs from the others in one of the four elements (toy, who, act and segment) and thus contributes to the count of the total number of selections by a dyad.

toy	who	act	segment
—	—	—	—
bucket	child	handle	*n*
hippo	parent	handle	*n*
hippo	parent	talk	*n*
hippo	child	handle	*n* + 1
giraffe	parent	handle	*n* + 1
hippo	child	handle	*n* + 2

## 3. Results

**Figure 2.**
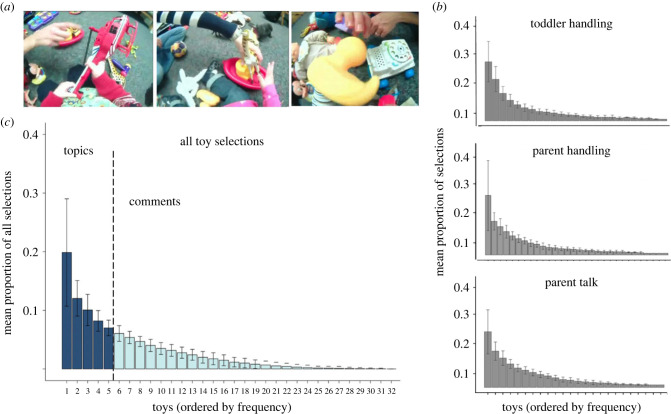
Toy selections. (*a*) Head-camera images of toy play. (*b*) Mean frequency of selection of individual toys (by rank order) normalized as mean proportion of within each selection behaviour: toddler handling, parent handling and parent referential talk. The horizontal axis shows unique toys by their rank frequency standard deviations. (*c*) The mean normalized frequency distribution for all unique toy selections as defined in table 1. The objects are partitioned into two categories: Topic objects (the five most frequently selected toys) and Comment objects (the more rarely selected toys). The error bars show the standard deviation. (Online version in colour.)

Figure 2*a* shows examples of toddler head-camera images during active play. Within 89% of the 5 s segments, at least one toy was selected by at least one of the three behaviours (range 0.35–1 s.d. = 0.25). Parents and toddlers handled objects roughly equally: the mean proportion of video segments with toy handling by toddlers was 0.86 (range 0.18–1, s.d. 0.43) and for parents, it was 0.70 (range 0.24–1, s.d. 0.33). Parent talk referenced at least one toy in 0.80 of all segments (range 0.51–0.97, s.d. = 0.18).

Despite this continued engagement with the toys, the dyads did not broadly sample the toys available in the room but instead were highly selective. The mean number of the unique toys handled at least once by the toddler was 15.2 (range 8–25, s.d. = 4.27) and by the parent was 17.4 (range 8–30, s.d. = 5.2). The mean number of unique objects referenced in parent talk was 16.5 (range 8–24, s.d. = 3.6). This selectivity was not due to all the dyads favouring the same specific toys. All 32 toys were selected by at least one dyad for play and 30 unique toys were among the top five most frequently selected toys for at least one dyad.

### (a) Frequency distributions

Figure 2*b* shows the mean frequency distributions of toy selections averaged not by the specific toy, but by rank order of selection. Frequency is normalized as the proportion of total toy selections for the participant and selective act. All selection acts—toddler handling, parent handling and parent talk—show similar distributional patterns: a few toys were frequently selected in play and many others were also selected but not frequently. We used two statistical tests to determine whether the three time series of selections by different behaviours differed and conducted both sets of analyses at the individual dyad level. The first test compares distributional properties using the Wilcoxon test for comparing whole frequency distributions of rank-order data, asking whether they show the same degree of skew, a characteristic property of behaviour-generated events. Within each dyad and for each of the three pairwise comparisons, we observed no reliable differences. For the comparison of infant and parent handling, the mean Wilcoxon V was 192, with the range across dyads of 23–310, and *p* > 0.01 for all individual dyads. For the frequency distributions of parent handling and parent talk, mean *V* = 186, range 63–280 and *p* > 0.01 for all the dyads. For the comparison of the frequency distributions of parent talk and infant handling, mean *V* = 190, range 39–327 and the *p*-value > 0.01 for all dyads. In brief, for all selection acts and all participants, a few toys were selected many times and many toys were selected a few times.

The second set of tests asked whether, within dyad, the different selection behaviours selected the same toys with the same frequency. To this end, we used Spearman rank order correlation, measuring the correspondence in rank order of the specific toys selected by different behaviours. All separately conducted dyad correlations (d.f. = 31 of the 32 possible toys) were reliable at *p* < 0.01: the mean correlation in rank order selection by toddler and parent handling was 0.64 (range 0.54–0.96 across dyads); 0.71 (range 0.60–0.92) between parent talk and toddler handling; and 0.72 (range 0.60–0.93) between parent talk and parent handling. These findings tell us that the frequency distributions of individual toys by the three selection acts within a dyad were highly correlated.

The histogram shown in figure 2*c* is made by counting all the forms of toy selections within each dyad to the same toy. Using the sample data in table 1, we illustrate the count entries to the same toy in the 'toy' column (four hippo events, one bucket event and one giraffe event). We use this aggregate count of selected toys (ignoring who and the specific behaviour) in the following analyses. This approach is justified on four grounds: (1) the lack of distributional differences and strong correlations within each dyad in the frequency with which specific toys were selected; (2) the series of all acts, *captured from the point of view of the toddler*, is the time series of toy selections experienced by the toddler; (3) in studies of parent–infant play [40,41], handling of a toy by parent or infant, as well as parent talk about a toy, is often conceptualized as a deictic act that refers to the handled entity [40,41]; and, thus, (4) the combined time series represents the overall joint structure of the emerging ‘conversation’ of play.

### (b) Topics and comments

Theorists of narratives attribute different informational roles to the high- and low-frequency components of the narrative [29,35]. We borrowed the information-theoretic definitions [35] of Topics and Comments to partition each toddler's experienced time series of toy selections into two categories: the five most frequently selected toys versus the other toys. The top five toys accounted, on average, for slightly more than 50% of all toy selections (mean = 57%, range 43–82%, s.d. 11%). In discourse [32–35,40], references to the main topics are characterized by close-in-time repetitions and distant-in-time repetitions (figure 1*b*). Returns to the main Topics after longer delays hold the narrative together, connecting old and new content [32,33,36]. Does this pattern also characterize the time series of parent–toddler play?

Close-in-time repetitions and returns over longer delays could emerge during play solely from the already documented frequency distributions; high-frequency selections of a toy provide a greater likelihood of both close and far repetitions than low-frequency selections. To determine whether the observed temporal patterns are stronger than those expected solely by the skewed frequency distributions, we created for each dyad a randomly permuted series of all toy selections that disrupted the temporal properties of the times series but maintained the frequency distribution of the individual toy selections generated by the dyad. For each dyad, 10 000 Random permutations of the observed time series were used to create the random baseline (see [34] for a similar approach). The randomly permuted time-series instantiate the null hypothesis that the measured statistics emerge solely from the observed frequencies of the selections of the different toys. The alternative idea is that the processes that create moment-to-moment selections are dynamically related in ways that create coherence beyond what is expected to emerge from the skewed frequency distribution alone.

To examine the close-in-time repetitions of Topics and Comments—which is sometimes referred to ‘continuity’ in spoken discourse [34]—we defined a cluster of selections of the same toy as a *minimum* of two selections of that toy that occurred in *adjacent* segments. That is, for these cluster measures, we count *runs of segments* with selections of the same toy (but not number of selections of the same toy in the same segment). Using the example data in table 1, there is a three-segment cluster of repetitions for selection of the toy 'hippo' because hippo is selected at least once in each of the segments *n*, *n* + 1 and *n* + 2. For each dyad, we computed the number of clusters and the run length of the cluster. For the example in table 1 (assuming hippo was not selected in segment *n* − 1 or *n* + 3), there is a single cluster of hippo with a run length of three segments. Table 2 shows the data for clusters and run lengths separately for the Topic and the Comment toys. The mean number of clustered repetitions was significantly *less* for the observed than randomly permuted data (*F*_1,31_ = 78.42 *p* < 0.01). There was also a main effect of Topic and Comment (*F*_1,31,_ = 63.2 *p* < 0.01) and a reliable interaction between the factors of observed-random and topic-comments (*F*_1,31_ = 37.6 *p* < 0.01). As shown in table 2, the observed time series results in fewer clusters because individual clusters have longer run lengths in the observed than in the random data. Toddlers and parents continue selecting the same toy for longer spans of play than would be expected by the frequency of toy selection alone. Thus, the mean run lengths of observed clusters were longer than the permuted baselines (*F*_1,31_ = 67.58 *p* < 0.01). Mean run lengths were also longer for topics than comments (*F*_1,31_ = 74.5 *p* < 0.01). The interaction approached significance (*F*_1,31_ = 5.76 *p* = 0.02). In brief, toy selection shows longer spans of play (segment runs) involving the same toy than would be expected from the frequency of the toy's selection and this is somewhat more marked for the topic than the comment objects.

**Table 2.** The mean number and run length of clusters of toy selections to the same toy. Objects at the Dyad level for the Observed times series of data and for the 10 000 Random permutations of the Observed times series. Standard deviations are in parentheses. A cluster is defined as at least two successive 5 s segments containing a referential act (toddler handling, parent handling and parent talk) to an object. The duration (in seconds) is calculated as the number of segments × 5 s. The cluster statistics were calculated separately for the high-frequency Topic objects and the low-frequency Comment objects. The component measures for Toddler Referential acts (handling) and Parent Referential acts (handling and talk) are given the Dyad-level data.

	topic	comment
**number of clusters**		
observed dyad	3.57 (1.9)	1.46 (0.78)
random dyad	15.82 (7.6)	4.09 (1.35)
**run lengths (number segments in a row)**		
observed dyad	4.82 (2.31)	2.63 (1.39)
random dyad	2.32 (1.82)	1.02 (1.24)

**Figure 3.**
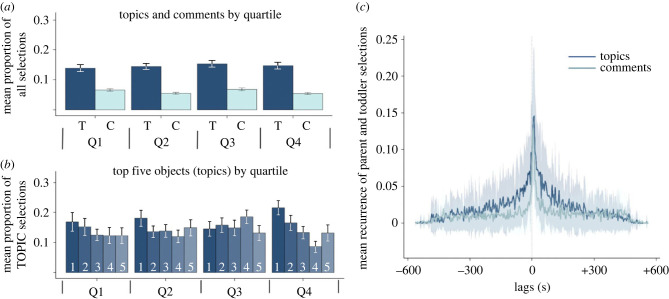
Temporal statistics of high-frequency Topic toy selections and low-frequency Comment selections. (*a*) The mean normalized frequency (proportion of all selections) Topic (darker) and Comment (lighter) toys for the four quarters of the 10 min play period. The bars show the standard deviations around the means. (*b*) The mean normalized frequency (proportion of all Topic selections in a quartile) for the five unique (by rank order) Topic objects for each quartile. (*c*) The mean cross-recurrence for parent toy selections (handling and talk selection included) and infant selections (handling) for Topic (darker) and Comment objects (lighter). The lighter bands show standard deviations at each lag. Positive lags indicate toddler selections that preceded parent selections of the same object. Negative lags indicate parent selections that preceded infant selections of the same object. (Online version in colour.)

Coherent discourse—our guide to measuring the coherence statistics of play—is characterized not just by the local continuity of topics but also by returns to the main themes over longer delays. We measured returns to previously selected toys over the course of the whole play episode by partitioning the play session into four quartiles (2.5 min in duration). Within each quartile, each dyad's score was the normalized frequency (that is, proportion of Topic selections divided by 5 for all dyads and the proportion of Comment selections divided by the number of non-topic objects referenced by the dyad). Figure 3*a* shows the mean normalized proportions within each quartile. A two-way repeated measures ANOVA revealed only a main effect of Topics and Comments (*F*_1,31_ = 80.29, *p* < 0.0001), with the frequency of Topic toy selections greater than Comment toy selections. There was no effect of Quartile (*F*_3,93_ = 0.80 *p* = 0.49) and the interaction between Topics/Comments and Quartile was not reliable (*F*_3,93_ = 2.19, *p* = 0.09). In brief, the mean proportion of referential acts directed to the Topic objects, considered in aggregate, did not differ across quantiles (*F*_3,31_ = 0.22, *p* = 0.89), showing that Topic objects recurred throughout the whole play session intermingled with the more numerous and variable Comment objects. Figure 3*b* shows the data separately for the top five objects (for each dyad) that comprise the set of Topic objects for that dyad. Each of the high-frequency Topic toys was selected in each quartile and thus the pattern in figure 3*a* is not due to different Topic toys being selected in different quartiles. These analyses show that the selections of high- versus low-frequency toys display a temporal pattern that is similar to the pattern of referential acts in discourse: the string of toys engaged by the dyad consists of frequent and repeated clustered ‘references’ to the same ‘main-theme’ toys interleaved with ‘references’ to new toys.

Although our main focus is on the statistics of toy selection that the toddler experiences and not on the relative role of the two participants in creating these statistics, their relative roles are a question of interest in developmental science [41–43]. It could be, for example, that parents have a sense of narratives and the parent scaffolds the toddler's play and is responsible for the observed pattern. To address this issue, we used cross-recurrence quantification [48,49], measuring the leads and lags in toddler contributions to toy selection (handling) and parent contributions to toy selection (handling and talk). We calculated the time-lags between toddler and parent selection of the same toy: the lag is zero if both parent and toddler selected that toy in the same segment; the lag is 5 s if parent and infant selected the same toy in adjacent segments, and the calculated lag for all other selections is the difference between the timestamps multiplied by 5. Cross recurrence determines *all* lags in time between all infant and parent acts on the same toy: if the infant held the object in segment 1 and the parent selected the object in segments 2, 5 and 120, the lags between 1,2 and 1,5 and 1,120 would all be calculated [49]. Positive values indicate toddler toy selections that preceded parent selections of the same toy; negative values indicate parent selections that preceded toddler selection of the same toy; zero indicates referential acts with the same time stamp (occurred in the same 5 s segment). Figure 3*c* shows the mean proportion of all lags between parent and infant selections of the same toy and also shows them separately for selections of Topic and Comment toys. The peaks of the recurrence plots lie near zero, indicating that referential acts to the same toy from the two partners most often occurred close in time. However, the peak recurrence for both Topic and Comment objects lies slightly but reliably on the positive side of zero, indicating that toddlers lead more often than parents. Across both Topic and Comment objects, there were on average more positive lags (55%; s.d. = 0.07) than negative lags (*t*_31_ = 5.2, *p* < 0.01). The mean lag for Topics objects was + 2.63 s (s.d. = 0.58) and for Comment objects was + 40.4 s (s.d. = 66). Thus, parents more quickly followed toddler interest to the same toy when it was a high-frequency Topic toy than a lower-frequency Comment toy. The main finding from the cross-recurrence analysis for the present purposes, however, is that parent and toddler toy selections are highly coordinated in time. The observed patterns are not created primarily by the parent but appear to be the joint creation of toddler and parent.

### (c) Higher-order structure

In studies of human-generated behaviours more generally, skewed frequency distributions and burstiness yield to small-world networks in which the unique component events are integrated into a single network by relatively few connecting edges [12,50]. Does toy selection during play have this same structure? We used graph-theoretic analyses to ask whether the time series of toy selection within individual dyads showed relational patterns. The analysis takes as data *all* the *pairs* of unique toy selections that occurred in the same segment (co-occurred) and in adjacent segments as shown in table 3 for the sample data in table 1. For simplicity, we will call both co-occurring (same segment) and adjacent (adjacent segment) pairs of unique selection acts *transitions* because they are close in time (same or adjacent segment) events. The transitions, so defined, fall into two classes: S transitions (pairs of the selections of the same object) and D transitions (pairs of selections of different toys). For example, using the data in table 3, bucket–hippo is a D pair regardless of who or how the pairing is made and hippo–hippo is an S pair and because pairs must be unique selective acts, the hippo–hippo pair must be selected by different people, different acts, or different (adjacent) segments. In our analyses, all edge connections were treated as non-directional; that is, when making and measuring the networks, giraffe–hippo and hippo–giraffe were treated as two instances of the same edge.

**Table 3.** The input to the network analyses consisted of pairs of co-occurring (same time stamp) or adjacent selections (successive time stamps). Shown are all the pairs that would be submitted to the network for the data shown in table 1.

bucket, hippo	(segment *n*, *n*: Child handle, Parent handle)
bucket, hippo	(segment *n*, *n*: Child handle, Parent talk)
hippo, hippo	(segment *n*, *n*: Parent handle, Parent talk)
bucket, hippo	(segment *n*, *n* + 1: Child handle to Child handle)
bucket, giraffe	(segment *n*, *n* + 1: Child handle, Parent handle)
hippo, hippo	(segment *n*, *n* + 1: Parent handle, Child handle)
hippo, giraffe	(segment *n*, *n* + 1: Parent handle, Parent handle)
hippo, hippo	(segment *n*, *n* + 1: Parent talk, Child handle)
hippo, giraffe	(segment *n*, *n* + 1: Parent talk, Parent handle)
hippo, giraffe	(segment *n* + 1, *n* + 1: Child handle, Parent handle)
giraffe, hippo	(segment *n*, + 1 *n* + 2: Parent handle, Child handle)
hippo, hippo	(segment *n*, + 1 *n* + 2: Child handle, Child handle)

**Figure 4.**
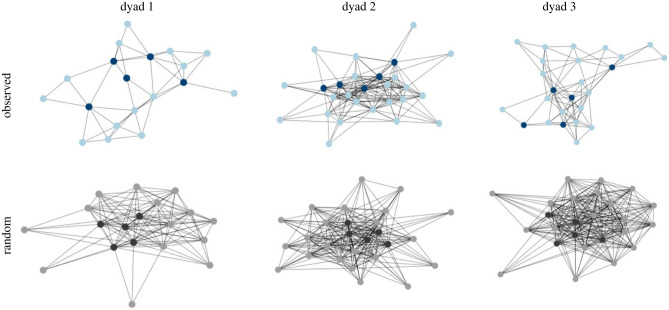
Networks of the transitions among different toys for three individual dyads and the random networks derived from random permutations of toy selections. All edges are non-directional and self-loops (S edges) are not shown. Proximity of nodes indicates the edge strength between them. Topic objects are shown as darker nodes than Comment objects. (Online version in colour.)

The individual dyad networks were constructed with each unique toy indicated as a node and transition pairs are indicated by the connecting non-directional edges. Figure 4 shows the networks for three individual dyads. For ease of visualization, we exclude the S transitions showing only the D transitions. We also show all edges that occurred at least once in the dataset; the frequency of an edge is indicated by the closeness of the nodes. In addition to the three Observed networks, figure 4 also shows the Random networks for each example network. Random networks were created from the mean transitions computed over the 10 000 random permutations of the order of each dyad's toy selections. Therefore, the Random networks instantiate the null hypothesis that the observed structure of transitions is solely the result of the frequencies of selection of individual toys.

**Figure 5.**
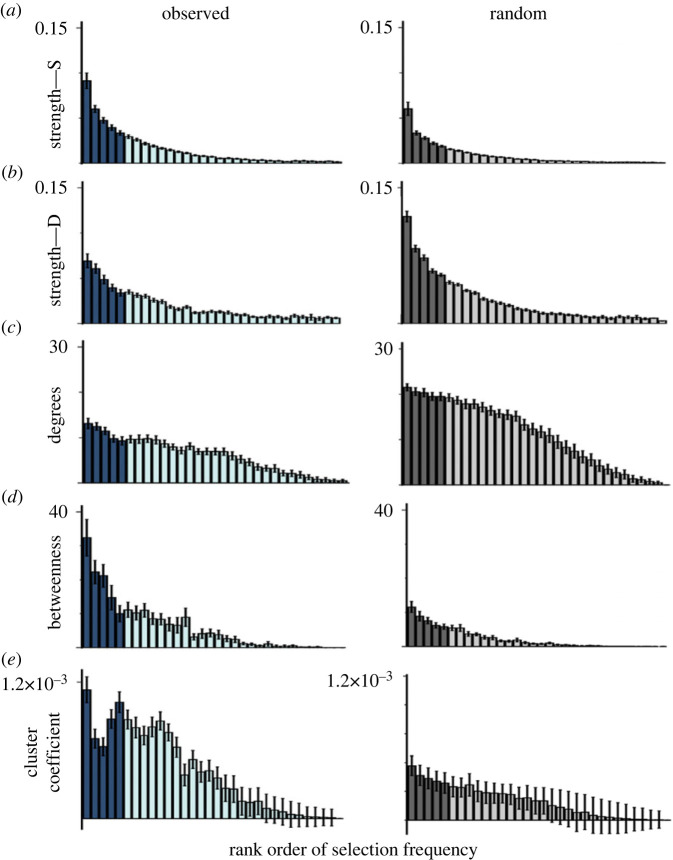
Statistics on network structure for Observed and Random networks. Each graph shows the measured statistic as a function of the rank order of individual toys by frequency of selection, with the frequent Topic objects shown in darker shades and the less frequent Comment objects shown in lighter shades. (*a*) Mean strength of the S-edges as a function of rank order of toy selection. (*b*) Mean strength of the D-edges as a function of rank order of toy selection. (*c*) Mean degrees, unique edges connecting to each node, by their rank order toy selection. (*d*) Mean between of each node by rank order of toy selection. (*e*) Mean cluster coefficient (transitivity) by rank order of toy selection. The error bars show the standard deviations around the means of each object, or node in the network. (Online version in colour.)

We used four measures of network structure [51] that were computed separately for each dyad's Observed and Random network in Matlab [52]. The formulae and code are available at: (doi:10.17605/OSF.IO/HE7TZ). Figure 5 shows the means for each measure determined separately for each dyad for each node in the Observed and the Random networks. Statistical analyses of the network measures used a 2(Observed-Random) × 2(Topic-Comment) analysis of variance with both Network type and Node type as within factors.

**(1) Edge strength** is a measure of the frequency of individual transitions. A node with high strength participates in relatively many strong edges. We computed edge strength separately for the S and D pairs. As shown in figure 5*a*, the nodes in the Observed networks have stronger (more) S loops than do the Random networks (*F*_1,31_ = 221.5, *p* < 0.001). Parents and toddlers often repeat the same toy selection, selecting the same toy in two adjacent segments or in the same segment but by different selection acts. Topic objects had stronger S loops than did Comment objects (*F*_1,31_ = 159.7, *p* < 0.001); the reliable interaction indicates that the greater strength of S edges for Topics was more pronounced for the Observed than Randomly permuted data (*F*_1,31_ = 150.7, *p* < 0.001), indicating that the greater strength of self-loops for Topics over Comments is not simply the result of the greater frequency of selecting Topic toys.

The strength of edges connecting different toys—D edges—showed the inverse pattern (figure 5*b*). The strength of D edges was significantly greater in the Randomly permuted network than the Observed network (*F*_1,31_ = 184.9, *p* < 0.001). D transitions were also greater for Topic than Comments (*F*_1,31_ = 368.4, *p* < 0.001), and the interaction was reliable (*F*_1,31_ = 68.28, *p* < 0.001). A well-structured network connects nodes to each other with *fewer* distinct edges; thus, the fewer D edges for the Observed than Random networks implicates more selective structure.

(2) **Degrees** measures the number of unique edges connecting to a node. As apparent in the example networks in figure 4 and in the measures of all dyad networks in figure 5*c*, the nodes in the Observed networks connect much more selectively than is the case for the Random networks (*F*_1,31_ = 310.16, *p* < 0.001), in which individual nodes connect more indiscriminately to other nodes. That is, the observed networks connect all played-with toys with a few of the possible transitions that could have occurred given the observed frequency distributions of toy selection. This result is another marker of the coherence of toy selections; transitions between two different toys are often repeated. Topic nodes have more degrees than Comment nodes (*F*_1,31_ = 2626.76, *p* < 0.001), a difference that is not due to frequency effects alone as indicated by the reliable interaction (*F*_1,31_ = 136.661, *p* < 0.001). Selection of a Topic toy is often paired with the selection of other Topic toys *but also with the selection of rarer Comment toys* and this is not due simply to the greater frequency of Topic toy selections. In brief, the observed networks of selections represent a limited number of those that are possible given the observed frequency distribution. Moreover, Topic toys do more of the work of connecting different toy selections than would be expected by their higher frequency alone.

(3) **Betweenness** measures the number of times each node appears on a shortest path between two other nodes in the network. For example, if the time series contains the pair giraffe–boat and the pair cow–boat but does not contain the pair giraffe–cow, then boat would be on the shortest (three-node) path connecting giraffe and cow in the network. Nodes that have high betweenness play a greater role in determining network structure and are a signal of higher-order structure (that is, higher than pairwise transitions). By this measure (figure 5*d*), Topic toys play a greater role in structuring the time series of toy selection than would be expected by the greater frequency of Topic toy selections. Betweenness was greater for Observed than Random networks (*F*_1,31_ = 71.292, *p* < 0.001) and for Topic than Comment nodes (*F*_1,31_ = 130.985, *p* < 0.001), and the interaction was also reliable (*F*_1,31_ = 43.860, *p* < 0.001). Topic toys fall between the selection of other toys and thus connect those other toys to each other.

(4) The **Cluster coefficient** measures the transitivity of pairs in a network, an additional measure of higher-order structure. For example, if the time series contains the pairs giraffe–boat and the pair cow–boat and also contains the pair giraffe–cow, then the pairs are transitive and appear as a triangle pattern in the network (figure 4). As shown in figure 5*e*, this higher-order structure systematically characterizes the Observed but not the Random networks. The Cluster coefficient was greater for the Observed than Random networks (*F*_1,31_ = 35.479, *p* < 0.001), for Topics than Comments (*F*_1,31_ = 34.767, *p* < 0.001); and the interaction was reliable (*F*_1,31_ = 9.226, *p* < 0.001). As can be seen in figure 5*e*, the Cluster Coefficient is much greater in the Observed data for both the Topics and Comments than the Random networks. This indicates that transitivity—a higher-order coherence beyond the selections of individual toys or pairs of toys—includes both Topic and Comment toys.

The network analyses show that parent and toddler toy selections are not a string of unrelated events but have selective and recurrent relational patterns centred on a few central toys. In brief, the time series of toy selections have the statistical properties that theorists of narratives point to as critical to integrated and coherent meaning.

The time series of toy selections by parents and their toddlers are not true narratives in the usual sense and the underlying semantics are not of the usual kind one thinks about in the context of discourse or stories. More specifically, the edges connecting pairs are not typically taxonomic (hippo–giraffe) or thematic (truck, hippo riding), although those relations do sometimes occur. For example, one toddler's transitions often included banging objects—banging the cow with a hammer *and* banging the cow with the giraffe (used and held like a hammer) and both of these hammering acts occurred in both clustered repetitions and in returns after longer delays. For another toddler, there were repeated transitions—S and D edges—of putting toys in, out, on and off other things, including on an upside-down (wheels up) truck. For others, there were series of fingerings of like features on a succession of different toys—fingering wheels on several vehicles, or eyes on several animals. There were many other *idiosyncratic* patterns that in retrospect make sense as explorations of the toys. Thus, the relational patterns are not like a story with a plot that an adult would tell or recognize. But the time series of toy selections are story-like in their temporal statistics: there are a few ‘protagonists’ (the Topic toys) that play a major role throughout play, that have ‘adventures’ with other the less-frequent toys, and that connect all the selected toys in a network of repeated relational patterns. These idiosyncratic but structured patterns of toy selection emerged in a context of free play with minimal contextual constraints on how parents and toddlers should sample the toys. What created these coherence statistics in toy play? What do the observed coherence statistics mean for cognitive development?

## 4. General discussion

### (a) Generating the statistics

This suite of statistics observed in parent and toddler play is generally understood as emerging within complex systems that have a ‘memory’, such that each generated event influences the likelihood of specific future events [50]. If parent and toddler play had no ‘memory’ for what had happened previously in play, but only had persistent biases for some toys over others, then the measured statistics would not have differed from those of the randomly permuted time series. The continuity of the Topics across short and longer time spans, the selectivity of transitions and the betweenness and transitivity patterns all implicate a role for memories—for both toddler and parent—of previous toy selections on play.

One relevant memory process is the transient shorter-term memories often known as working memory, although contemporary understanding differs substantially from classic views of working memory as a limited number of slots with a set decay rate [53–58]. Recent findings about the neural underpinnings of working memory reveal that in-task transient memories exist as a kind of ‘long-range recurrent feedback loop’ [53,54] across multiple neural networks involving perception, attention, action and planning. Experimental evidence shows these transient memories to be less time- and content-limited and more resistant to interference from momentary shifts in attention than did classic views and to hold information in both an active state and in a silent but ready-to-be-re-activated state [53–55]. In these accounts, working memory is task-length; it stores associative and predictive relations among events and reactivates and strengthens those relations through pattern completion [53–56]. If these processes operate within parents and toddlers to generate toy selections then one should see returns to frequent toys, even when interleaved with other toy selections, with each selection of a toy increasing the likelihood of selecting that toy again. Because working memory stores and strengthens predictive relations, play with one toy should serve as a reminder—through pattern completion—of play with a different toy that previously preceded or followed that toy in play. These specific toy-to-toy transitions should also strengthen over the play session. These within-episode memories may also support exploration and innovation within the play session. For example, if a toddler has formed a predictive association between banging the cow (with the hammer) then looking at the cow while holding the giraffe may reactivate that association and lead to banging the cow with the giraffe by holding the neck of the giraffe as the handle of a hammer. Thus, the properties of task-length transient memories have the potential to generate the statistics observed here: fostering repetition and returning to previous topics over short and long lags, leading to generalization and innovation (e.g. banging cows with giraffes). At present these are conjectures, but they suggest the agenda for future research: the complex dynamic processes of working memory in extended episodes of parent–toddler joint play are a target mechanism for generating the observed statistics.

Physical space also has a ‘memory’. During the play session, the toddlers were free to move and often did so to retrieve an out-of-reach toy. However, children and also parents when transitioning from one toy to the next often put handled toys down near their own bodies. Thus, at any moment, the spatial layout of toys near the two partners constituted an external ‘memory’ of toys that had been recently selected. Proximity makes these toys easier to see (re-activating memories) and easier to reach, exemplifying Zipf‘s [13] principle of least effort that yields a ‘more-gets-more’ dynamic in human activity.

However, these memory processes alone may not provide a complete explanation. Throughout play, parents and toddlers continually added new toy selections, creating what we called the Comment toys. A complete explanation of the observed statistics will need to integrate ‘more gets more’ processes with exploration, perhaps by linking contemporary theories of working memory to curiosity [14–20]. Much of human behaviour is characterized by the push and pull of the familiar and the novel [59,60], including phenomena such as habituation and dishabituation [61], prediction and surprise [62,63], exploitation and exploration [1]. The present findings implicate underlying processes that boost returns to prior toys and the intermittent and interleaved exploration of new toys. What drives this push and pull to return to the same toys and explore new ones? One possibility is the internal rhythms of attention [64,65]. Another possibility is the time-dependent processes of memory strengths and decay over time that have been known since Ebbinghaus [66]. A third possibility is suggested by current ideas about predictive coding [67,68]; in this view, the cognitive system is continually generating and updating a model of the world and does so by generating predictions, determining the prediction error and correcting the model. When prediction error diminishes, the learner moves on to novel problems [33,34]. Experimental studies of this phenomenon have concentrated on experimentally controlled procedures and discrete screen-based trials to test hypotheses [33,44] that reveal critical internal mechanisms. But is not clear how these processes might work at the timescale and complexity of everyday life. We conjecture that in everyday experience, prediction error may not be calculated across individual events (using a hammer on a cow) but rather across the whole emerging series of events (relations among hammers, giraffes, buckets and cows). From this perspective, the observed returns to familiar objects and the explorations of new ones may reflect a single *within-episode* trajectory of decreasing predictive error by increasing knowledge about the *relations* among all the selected toys. If banging works with a hammer, does it also work with a properly held (for hammering) giraffe? Can one properly hold a cow for banging things?

### (b) Learning from the statistics

The transient memory systems that we propose generate the observed statistics are likely also involved in learning from those statistics. Recent advances including both animal and human research (see [38,53,54] for reviews) indicate that durable and expressible memories form rapidly (within a single extended-in-time episode) under conditions in which: (1) the to-be-learned items emerge from previous actions and influence next actions; (2) the to-be-learned items are part of a social interaction; (3) the context is multimodal, recruiting recurrent activation across many neural networks and (4) when there are predictive relations among temporal order of the to-be-learned items. All these factors are believed to support the persistent, repeated and cued (through pattern completion) activations that have been shown to be central to the rapid formation of durable and retrievable memories [53,54,69,70]. These conditions are all present in the observed exploratory play by parents and toddlers. At every moment of the interaction each participant's selection of a toy emerges from a previous selection and influences the next. The context is social and multimodal—visual objects, heard sounds, planned actions, language and emotion. In brief, parent–toddler active toy play presents a learning environment characterized by a suite of statistics that create coherent episodes of experience and that is well matched to known optimal conditions for turning working memories into permanent ones.

### (c) Implications for conceptual development

We began this paper with a description of the statistics of human-generated experience. Children, from birth forward, develop in the company of others and in contexts temporally structured by the dynamics of human behaviour, and with development, increasingly structured by the child's own behaviour. Although there is much general interest in these statistics across many domains, they are not generally studied as a suite of interdependent statistics nor with respect to the statistics of human everyday experience. Most experiments on learning use brief training trials (seconds long) with random and uniform distributions of training experiences (but see [18–23]). In the current work, we looked to the study of narratives—not because play fits the formal properties of discourse, but because theorists of narratives have thought about the temporal statistics of human-generated events in terms of how those statistics convey coherent information. We showed that parent–toddler free-flowing play exhibits similar statistics and, by implication, episodic coherence.

The coherence is evident in the patterns of repetitions over short and long lags and in the transitions in toy selections. Like words in a discourse, or characters in a story, toy selections cohere into an integrated experience describable by a network. The observed networks serve as potentially useful hypotheses about the structure of internal memories formed during coherent episodes of experience. These memories may consist of more than a string of separate events, whole network of relations and predictive patterns. There is emerging experimental evidence that memories are readily formed of prediction relations and higher-order patterns in experienced time series [71]. If memories of episodes of experience are formed as networks of relations, then novice learners who may know little about the underlying principles of causes and effects in the world (both physical and social) may be able to discover them by statistically aggregating information not just within individual episodes of experience but across episodes. Seminal research on children's learning about the thematic structure of everyday events [72,73] and relational learning [74] offers empirical and theoretical foundations for pursuing this idea.

## 5. Conclusion

The sampling statistics of toy selection during parent and infant free-play shares temporal statistical properties with that of narratives. Episodes of toy play may be experienced and remembered, as are stories, as a coherent system of meaningful relations. If so, the suite of statistical properties observed here may play a critical role in powering early cognitive development.

## Data Availability

The data and code are available on the Open Science Framework: https://doi.org/10.17605/OSF.IO/HE7TZ [75]. The data are provided in electronic supplementary material [76].
